# A Woman's Experience in a Surgical Ward

**Published:** 1913-08-23

**Authors:** G. Beatrice Ruggles

**Affiliations:** (Patient in Drummond Ward, St. George's Hospital). 8 Swan Walk, Chelsea Embankment, S.W.


### THE EDITOR'S LETTER-BOX.

### "A Woman's Experience in a Surgical Ward."

*To the Editor of* The Hospital.

Sir,—It is with much surprise and regret that I read the letter from a patient, published in The Hospital for August 16.

I cannot refrain from asking the question, What hospital has she been in?

During the last seven years I have had much to do with trained nurses, and I cannot speak too highly of their work. I have also spent three weeks as a patient in a surgical ward, and, although this is two years ago, I often tell others with pleasure the kind care and attention I received from probationers, nurses, and sisters in the ward. I am much interested in this very good work, which is being done for our sick and suffering, and I naturally took much notice of the detail work. I so particularly noticed how carefully everything was taken to be sterilised, and the clinical thermometers were always kept in a glass of fluid disinfectant, and each one after being in use was always returned to this glass of fluid before being given to another patient. I could contradict everything this patient complains of. I never once felt a jar or heard a door bang, and our bedsteads all had rubber castors, so that when the bedstead was moved one scarcely felt it. Whenever I was awake during the night, which was very often, the nurse or probationer on duty came to ask if I was all right or in any pain. I can only come to the sad conclusion that this patient was not in one of our London hospitals, where one can truthfully say everything is well done.

I really feel that such letters as this patient sends will add to the dreadful impression many have of our hospitals.

—Yours faithfully, G. Beatrice Ruggles

(Patient in Drummond Ward,

St. George's Hospital).

8 Swan Walk, Chelsea Embankment, S.W.

[Our correspondent raises the question as to what kind of hospital provided the treatment which a patient describes in The Hospital of August 16, page 589, as a woman's experience in a surgical ward. In publishing various accounts of the hospitals from the patients' point of view we have purposely not given the names of the particular institutions referred to, our aim being to publish an unbiassed and unedited account of each patient's experiences, merely satisfying ourselves before publication that the contributions are *bona fide* and written fairly and impartially, being based on actual happenings. We may, however, state that the patient in question was not an inmate of a general hospital, but of a hospital which is established for the reception of women, and which is entirely administered and worked by women in every department.—Ed. The Hospital.]

